# Perspectives on the COVID-19 Vaccination Rollout in 17 Countries: Reflexive Thematic and Frequency Analysis Based on the Strengths, Weaknesses, Opportunities, and Threats (SWOT) Framework

**DOI:** 10.2196/44258

**Published:** 2024-02-19

**Authors:** Vanja Kopilaš, Khrystyna Nasadiuk, Lucia Martinelli, Lenka Lhotska, Zoran Todorovic, Matjaz Vidmar, Helena Machado, Anna Lydia Svalastog, Srećko Gajović

**Affiliations:** 1 Department of Psychology Faculty of Croatian Studies University of Zagreb Zagreb Croatia; 2 Department of Biochemistry Danylo Halytsky Lviv National Medical University Lviv Ukraine; 3 Science in Society Program MUSE-Science Museum Trento Italy; 4 Czech Institute of Informatics, Robotics and Cybernetics Czech Technical University in Prague Prague Czech Republic; 5 Faculty of Biomedical Engineering Czech Technical University in Prague Prague Czech Republic; 6 University Hospital Medical Center "Bežanijska kosa" Belgrade; 7 Department of Pharmacology, Clinical Pharmacology and Toxicology Faculty of Medicine University of Belgrade Belgrade; 8 Institute for the Study of Science, Technology and Innovation The University of Edinburgh Edinburgh United Kingdom; 9 School of Engineering The University of Edinburgh Edinburgh United Kingdom; 10 Institute for Social Sciences University of Minho Braga Portugal; 11 Østfold University College Halden Norway; 12 Centre for Research Ethics and Bioethics Uppsala University Uppsala Sweden; 13 BIMIS-Biomedical Research Center Šalata University of Zagreb School of Medicine Zagreb Croatia

**Keywords:** SARS-CoV-2 virus, COVID-19 vaccination, pandemic, hesitancy, safety, vaccination, COVID-19, tool, implementation, vaccine hesitancy, effectiveness, sociocultural, communication, disinformation

## Abstract

**Background:**

As the SARS-CoV-2 virus created a global pandemic and rapidly became an imminent threat to the health and lives of people worldwide, the need for a vaccine and its quick distribution among the population was evident. Due to the urgency, and on the back of international collaboration, vaccines were developed rapidly. However, vaccination rollouts showed different success rates in different countries and some also led to increased vaccine hesitancy.

**Objective:**

The aim of this study was to identify the role of information sharing and context sensitivity in various vaccination programs throughout the initial COVID-19 vaccination rollout in different countries. Moreover, we aimed to identify factors in national vaccination programs related to COVID-19 vaccine hesitancy, safety, and effectiveness. Toward this end, multidisciplinary and multinational opinions from members of the Navigating Knowledge Landscape (NKL) network were analyzed.

**Methods:**

From May to July 2021, 25 completed questionnaires from 27 NKL network members were collected. These contributors were from 17 different countries. The responses reflected the contributors’ subjective viewpoints on the status and details of the COVID-19 vaccination rollout in their countries. Contributors were asked to identify strengths, weaknesses, opportunities, and threats (ie, SWOT) of the respective vaccination programs. The responses were analyzed using reflexive thematic analysis, followed by frequency analysis of identified themes according to the represented countries.

**Results:**

The perspectives of NKL network members showed a link between organizational elements of the vaccination rollout and the accompanying societal response, both of which were related to strengths and weaknesses of the process. External sociocultural variables, improved public communication around vaccination-related issues, ethical controversies, and the spread of disinformation were the dominant themes related to opportunities and challenges. In the SWOT 2×2 matrix, *Availability* and *Barriers* emerged as internal categories, whereas *Transparent communication and promotion* and *Societal divide* emerged as key external categories.

**Conclusions:**

Inventory of themes and categories inspired by elements of the SWOT framework provides an informative multidisciplinary perspective for effective implementation of public health strategies in the battle against COVID-19 or any future pandemics of a similar nature.

## Introduction

On March 11, 2020, the World Health Organization declared the COVID-19 pandemic [1]. COVID-19 first appeared in December 2019 in Wuhan, China. This disease, caused by the SARS-CoV-2 virus, led to an unprecedented challenge for health institutions that required most countries to integrate their efforts to globally mitigate the spread of the disease [2-4].

Various policies to control the spread of the virus have been adopted in different countries. Some of them were drastic, such as national lockdowns, as well as initiating the widespread use of individual protection devices and means [5]. The individual protective measures included recommending frequent hand washing and application of sanitizers, maintaining social distance, and mandatory wearing of face masks or respirators. However, even a simple measure of covering the face proved to have psychological, cultural, religious, and behavioral implications at both the individual and communal levels [6]. Moreover, the policies aimed to stop the spread of the virus impacted the psychological well-being of the population [7]. These implications should be considered in public campaign strategies aimed at achieving effective public consent toward the adoption of protective measures.

The publication of the genetic sequence of SARS-CoV-2 on January 11, 2020, resulted in the explosion of comprehensive studies on the virus and stirred global research and development activity to develop vaccines against the virus [8]. To accelerate this work, next-generation vaccine technology platforms have been deployed and the first COVID-19 vaccine candidate entered human clinical testing as early as March 16, 2020. In December 2020—in record time and following collaborative efforts of the global scientific community, pharmaceutical companies, and governments—several types and brands of vaccines based on different technologies and mechanisms of action became available for mass deployment [9].

The importance of mass vaccination has been established in the context of previous epidemics such as in the case of smallpox eradication and the incidence reduction of measles and polio [10]. The goal of mass vaccination programs is to interrupt person-to-person disease transmission by surrounding infected people with a high proportion of vaccinated individuals who have developed protective antibodies against the infection (ie, reaching “herd immunity”) [11]. Public health experts have prioritized increased vaccination delivery with the hope to resume socioeconomic activities [12]. According to one study, to reduce the number of confirmed COVID-19 cases and deaths, it was estimated that, on average, the administration of 80 vaccine doses per 100 people was necessary [13]. However, the efficacy of vaccination is challenged by an increasing number of mutated strains, clinically proven possibilities of reinfection, and globally uneven rates of vaccination [14,15].

The vaccination process depends on various societal factors such as vaccine hesitancy, vaccine refusal, practical aspects of its application, and uneven/unequal vaccine rollout [16]. Even prior to the COVID-19 pandemic, the World Health Organization identified vaccine hesitancy as one of the top 10 global threats to public health [17]. After the appearance of COVID-19, this issue has gained a completely different dimension, and several people showed different degrees of vaccination acceptance from total refusal to hesitation, including health workers [18]. Levels of COVID-19 vaccine acceptance and obstacles to its rollout are country- and context-dependent [19]. Research has shown that most people are neither absolutely for nor against COVID-19 vaccines [20,21]. Hence, to begin to understand vaccine acceptance, it is important to gain insight into the reasons behind individual and collective decision-making [22].

SARS-CoV-2 is a novel virus, and various questions about dealing with this threat by mass vaccination emerged during the pandemic, including the efficacy of vaccines, the duration of the vaccine’s effect, and the impact of new virus variants [23]. The rapid vaccine development raised questions regarding safety, availability, and efficacy [24]. This is not surprising given the fact that vaccine development usually takes 10-15 years, whereas COVID-19 vaccines were developed in less than 1 year [25]. In addition, there are various factors that can increase disease spread and mortality rates that seem incoherent with the proposal for a uniform global vaccination rollout. The mortality rates were lower in countries investing more in the health system and vice versa [15,26]. Research from the United States showed that prosperous states with a higher population of older people and a higher number of physicians had a lower rate of vaccine hesitancy compared to that of other states [12]. The availability of vaccines, both in terms of the number of doses and equal distribution, has been an important issue within various countries, involving technical as well as socioeconomic aspects [27]. Timing is also very important since seasonal and environmental factors play important roles in the reduction of COVID-19 symptomatology [15,28]. Due to the numerous factors involved, interdisciplinary collaboration appears to be an appropriate solution to tackle vaccine hesitancy [29].

To facilitate a discussion on a successful vaccination rollout process, in this study, an analysis inspired by the strengths, weaknesses, opportunities, and threats (SWOT) framework was performed to explore perceptions and establish an informative perspective of the vaccination campaigns in 17 countries during the first phase of the mass vaccination programs in the first half of 2021. To facilitate this research, the scholars from the interdisciplinary Navigating Knowledge Landscape (NKL) research network were surveyed between May and July 2021. They were asked to provide information and their own opinions about the vaccination rollout programs in their respective countries. The participating scholars belonged to different disciplines, creating a specific combination of sociological and cultural analytical competences merged with medical and public health expertise. The aim of this interdisciplinary and transnational analysis was to better understand how information-sharing practices and social context were intertwined to coproduce public opinion on vaccination as a response to the COVID-19 pandemic.

SWOT, as a strategic planning framework, is usually used in evaluation of an organization, plan, project, or business activity. It is therefore a significant tool for situation analysis that helps managers identify organizational and environmental factors affecting performance and operations [30]. The framework can be used to identify favorable and unfavorable factors and conditions, solve current problems in a targeted manner, recognize the challenges and obstacles faced, and formulate strategic plans to guide scientific decisions [30-33]. The SWOT framework strives to offer a comprehensive, systematic, and accurate description of the scenario in which a topic is located [34]. SWOT analysis has two dimensions: internal and external. The internal dimension includes organizational factors focusing on strengths and weaknesses, whereas the external dimension includes environmental factors, namely opportunities and threats [30]. Since SWOT analysis is primarily used in organizational studies, our goal was to use its elements as a conceptual and narrative analysis tool where focus was placed on the intertwining viewpoints of social, political, and public health practices. A similar approach has already been applied as a research method in which aspects of the SWOT framework were used to yield more precise and organized data [35]. However, to date, a SWOT-based analysis of the COVID-19 vaccination campaign has only been reported for India and Zimbabwe [36-38]. Therefore, with this study, we aimed to offer a new transdisciplinary and multinational viewpoint of the vaccination process.

## Methods

### Study Design

The data set included 25 contributions from 27 members of the NKL research network, collected from May to July 2021. These members contributed their viewpoints through a questionnaire aimed at mapping, in a representative manner, the rollout of the vaccination campaigns against SARS-CoV-2 during the early stages when vaccines were available to the general public.

All contributions were collected in a public data set, which is available with open access in Mendeley Data [39].

### Study Sample

The 27 contributors were from 17 different countries: Australia, Austria, Croatia, Czech Republic, Germany, Hungary, Italy, Norway, Portugal, Romania, Serbia, Slovenia, South Korea, Sweden, Turkey, Ukraine, and the United Kingdom (including England and Scotland). Three contributions from the same country were received from Slovenia, Sweden, and Portugal; two from Croatia and the United Kingdom; and one contribution from each of the rest of the countries. Two contributions were coauthored (from Australia and Sweden). The contributors come from different academic backgrounds, but most of them are experts in the fields of life sciences, sociology, philosophy, and medicine. However, it is important to note that the contributors were expressing their own opinions and perceptions.

### Measures

The questionnaire contained three parts asking about the status and details of COVID-19 vaccination in the respondent’s country. Contributors were asked to return a short-text (ie, narrative) answer of 200-300 words per part. In this study, we focused only on the SWOT-related aspect of the responses (ie, Part 1) and the responses to the other parts of the questionnaire (Parts 2 and 3) were considered only to identify the eventual contribution to the SWOT-inspired analysis. SWOT elements were selected among the entire response text during the analysis process. The specific questions are presented in Textbox 1.

**Questionnaire items.**

- Part 1: The national vaccination program

Describe the COVID-19 vaccination program in your country: what were its strengths, main weaknesses, opportunities to improve it, and threats to its success?

- Part 2: Public discourse and ethics

How would you describe public responses to your country’s vaccination program? What is your impression on the various collective attitudes toward the vaccination program in your country? Were there any ethical issues or concerns around the vaccine program in your country?

- Part 3: Personal experience

What is your personal experience, opinion, or attitude regarding COVID-19 vaccination?

### Ethical Approval

Ethical approval for this study was obtained from The University of Edinburgh, Scotland, United Kingdom.

### Data Analysis Procedure

To fulfill the study’s aims and obtain results, reflexive thematic analysis [40] and descriptive statistics (frequency analysis) of the themes were performed. This method is considered appropriate for exploratory research such as our study. Moreover, flexibility of the thematic analysis and opportunity for theme development seemed a great fit and application for our data set [40,41]. The open-ended questions allowed for formulating responses that enabled the respondents to frame the description of the vaccination process in their countries according to their own personal views.

For the purposes of reflexive thematic analysis, we divided the responses into four categories according to the elements of the SWOT framework. The subcategories of each category were identified and a list of the themes for each SWOT element was established. In a subsequent step, we analyzed the data for patterns and recurring topics. We looked for country-specific differences and similarities in regulations and practices. In addition, close attention was paid to how the experts made sense of their experiences with the vaccination process and how the issues addressed were expressed. In presentation of the research results, focus was placed on themes identified throughout the reflexive thematic analysis. The results were then contextualized based on the existing literature.

Following that, frequency analysis of the identified themes was performed in relation to the corresponding countries. In the case of multiple contributions from the same country referring to the same theme or subtheme, only one data point was counted. The obtained results are presented in the form of tables and graphs.

## Results

### Thematic Analysis

#### Overview of Themes

**Figure 1:**
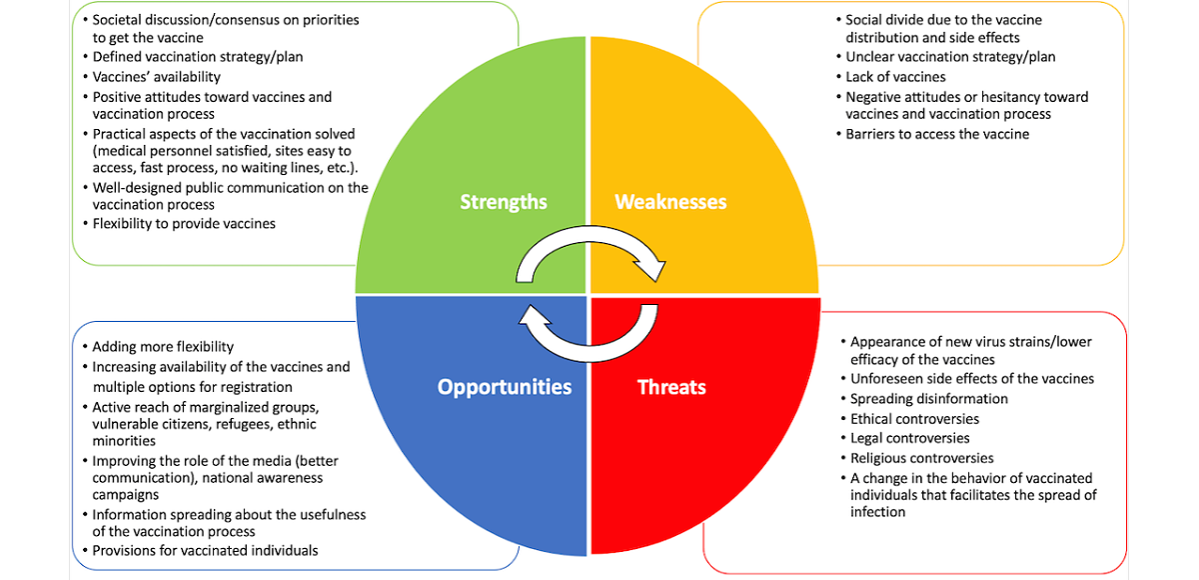
Graphical presentation of the established themes within each of the strengths, weaknesses, opportunities, and threats (SWOT) elements.

Reflexive thematic analysis of collected contributions was performed independently by two researchers (VK, KN). Through the process of the reflexive thematic analysis [40], the numbers of themes respectively belonging to the elements of strengths, weaknesses, opportunities, and threats were established (Figure 1).

Thematic analysis of the vaccination process yielded a nearly even distribution of the four SWOT elements across all included countries and contributors, with 7 themes identified for strengths, 5 themes identified for weaknesses, 6 themes identified for opportunities, and 7 themes identified for threats. In total, analysis of the SWOT elements covered 25 different themes.

The contributors shared their subjective perceptions of the effectiveness of the vaccination campaigns in their countries, which ranged from claims of success to voices of criticism. The United Kingdom was the first country in the world to start the COVID-19 vaccination program in December 2020. Shortly afterward, the vaccine rollout was launched in the United States and the countries of the European Union, albeit with some delay (3 months) in Ukraine. In many countries represented in this study, the vaccination rollout started with some constraints, poor planning/management, and delays with vaccines delivery, but improved over time. In Portugal, an efficient organization of the vaccination process was achieved with the change of the Head of the Vaccination Task Force. In the countries of the European Union, the vaccination process was coordinated with that of other member countries (Croatia), although this collaboration was not always perceived as efficient, as pointed out by a contributor from Sweden.

A successful vaccination program was achieved in the United Kingdom, being respectively described as “overall…a large success” and “an overwhelming success” [39]. The contributions from Portugal and Serbia highly rated the results of the vaccination programs in their respective countries in relation to the high vaccination rate and being ahead of plans/schedule. A relatively successful vaccination process was also reported in Croatia, Hungary, Italy, and Norway. Efficient implementation was noted in Turkey, and an active vaccination process was described in South Korea with major public facilities offering vaccinators discounts or exemptions from paying admission or usage fees. For some other countries, the collected contributions reported low vaccination coverage in the survey period (May to July 2021), including Australia, Romania, and Ukraine. The respondent in Romania specifically reported low coverage for high-risk groups and people over 65 years old. Moreover, very low coverage of rural areas occurred due to lack of local community involvement, especially mayors, policy makers, and family doctors, with some of the latter refusing to dispense vaccines.

Slow rollout of the vaccines was noted in Australia, Austria, and Germany. In Australia, the delayed vaccine rollout has been described as a “vaccine stroll out,” as by July 2021, only 6% of the Australian population had been vaccinated [39]. Moreover, some individuals in priority groups such as older people or those with disabilities living in long-term care homes were still waiting for their second or even their first dose of the vaccine. In addition, in some countries, the vaccination points were hard to access in remote, rural areas (Australia).

If we are to judge vaccination rollout success by looking at the percentage of people who had received at least one dose of the vaccine during the time period corresponding to our data collection, the most successful country in our sample was the United Kingdom, with approximately 70% of the population receiving at least one dose (Multimedia Appendix 1) [42]. The lowest percentage was reported in Ukraine, where only approximately 8% of people had received a single vaccine dose [42].

#### Strengths

The primary themes related to strengths included (1) societal discussion/consensus on priorities to get the vaccine, (2) defined vaccination strategy/plan, (3) vaccine availability, (4) positive attitudes toward vaccines and the vaccination process, (5) practical aspects of the vaccination solved (eg, medical personnel satisfied, sites easy to access, fast process, no long queues), (6) well-designed public communication campaign on the vaccination process, and (7) flexibility to provide vaccines.

High availability of vaccines was reported in Hungary, Italy, Sweden, and the United Kingdom. Following the controversies around the possible side effects of AstraZeneca vaccines, stocks of the European Union–approved vaccines were excessive in Slovenia. The wide availability of vaccines to whoever wanted them was considered a strength of the vaccination campaigns. In Romania, free vaccination has been offered to everybody who wanted one, including those with Romanian or European citizenship. In Portugal, vaccination was available independent of legal status, including to undocumented migrants. Free vaccination was also offered in Serbia to people from abroad, primarily citizens of neighboring countries, with no restrictions.

Medical workers played a key role in achieving successful vaccination campaigns. Family doctors contributed to the success of the vaccine rollout in Croatia and a helpful approach was reported by the medical staff of the Czech Republic. For Portugal, strong commitment of health care professionals and communication initiatives of the medical doctors to clarify doubts related to the vaccine’s side effects were noted.

Transparent planning and strategies, as well as prioritization of people with higher infection risk or greater vulnerability, were commonly reported strengths of the vaccination programs. In most countries, the prioritization was perceived as fair, although in some countries controversial cases of people from nonpriority groups being vaccinated early also occurred (Portugal, Slovenia). The priority groups in most countries included older adults, those with underlying health conditions, and workers exposed to a high infection risk. By contrast, vaccination of health care professionals has not been prioritized in Sweden. In all countries, the vaccine was provided free of charge, dispensed on a voluntary basis; however, mandatory vaccination was reported for medical workers in Italy and South Korea and for people in high-risk jobs in Australia. Moreover, an easy registration process, owing to easy-to-access platforms such as apps, web pages, or via the phone, was described for Turkey and Ukraine. Automatic enrollment based on medical records via general practitioners (eg, family doctors) was available in the United Kingdom. An efficient registration process in the Czech Republic was also claimed as a strength.

#### Weaknesses

The primary themes related to weaknesses were as follows: (1) social divide due to the vaccine distribution and side effects, (2) unclear vaccination strategy/plan, (3) lack of vaccines, (4) negative attitudes or hesitancy toward vaccines and the vaccination process, and (5) barriers to access vaccines.

Lack and shortage of vaccines were emphasized in Ukraine and Turkey, as well as at the beginning of the vaccination rollout in some other countries, where delayed deliveries were also reported. Delayed rollout to the remote Aboriginal communities was noted in Australia. The registration process was essential in achieving an effective rollout of the vaccines. Poor functioning of the distribution organization was highlighted by many participants from different countries, especially in the early stages of vaccination programs. Getting a vaccination appointment was rated as difficult in Sweden.

Trust was pointed out as an important issue in several contributions. A low level of trust in the medical science (Croatia), in the effectiveness and safety of the vaccines (Ukraine, Romania, Slovenia), and in the official authorities (Slovenia) were reported. An overall high level of skepticism in society at large was observed (Germany). Lack of enthusiasm and willingness to be vaccinated or vaccine hesitancy were widely reported (Austria, Norway, Romania, Slovenia, Ukraine). Despite the very successful vaccination process in Serbia, only a small percentage of younger people and health workers were vaccinated in the country. High hesitation among young people was also reported in Slovenia. In contrast, in Ukraine, young people were rather eager to be vaccinated, despite the high level of general hesitancy noted in the country. In Australia, vaccine hesitancy was exacerbated by the risks of side effects reported for the AstraZeneca vaccine.

Lack of clear and coherent communication on how to receive the vaccination was considered an important barrier to access in Slovenia. The need for suitable and unequivocal guidelines about vaccination was stressed in Italy, as constant changes have confused the population and discouraged vaccination, while different rules in different parts of the country and frequent regulation changes were noted to have discouraged vaccination in Germany. Unavailability of scientific information to foreigners/migrants, especially for those not fluent in the local language (eg, the home workers caring for the older population) was stressed for Italy. The digitally based vaccination approach was considered an important barrier to those not having adequate digital abilities. In Sweden, despite having one of the highest internet coverage rates in the world, people living in socially disadvantaged areas, including asylum seekers and migrants, and older adults or people with cognitive impairment who did not master the digital skills required were at risk to be excluded from accessing important information. A low level of digitalization was also mentioned as an obstacle to vaccination success in Romania.

#### Opportunities

The primary themes related to opportunities were as follows: (1) adding more flexibility; (2) increasing vaccine availability and multiple options for registration; (3) active outreach to marginalized groups, vulnerable citizens, refugees, and ethnic minorities; (4) improving the role of the media (better communication) and national awareness campaigns; (5) information sharing about the usefulness of the vaccination process; and (6) provisions for vaccinated individuals.

The freedom to choose to make an appointment for vaccination, no matter where people were registered (Sweden), and adding more flexibility to accessing vaccination (Croatia) were considered among the opportunities to improve the vaccination rollout.

To motivate people to be vaccinated, financial support (approximately US $30) was offered in Serbia. Vaccination coupons or exemptions from admission or usage fees of public facilities (approximately US $900) were introduced by a National Vaccine Injury Compensation Program in South Korea. In addition, this country also allowed a one-day “vaccination leave” from work to be taken the day after receiving the vaccine, along with an additional one-day leave in the case of experiencing some subsequent side effects [39]. In Ukraine, in the unlikely case that vaccination would cause disability or death, a compensation allowance (approximately US $21,000-27,000) was promised by the government.

Among the opportunities to improve the vaccination process, the freedom to choose among the available vaccine brands/types was recognized as a good strategy to counteract the arising doubts about a certain brand of vaccine (Slovenia, Ukraine). The choice of vaccine brand was also available in Turkey and in Serbia, contributing to successful vaccination campaigns. Finally, a more responsible role of media was mentioned as an opportunity to improve people’s attitude toward vaccination, as pointed out for Croatia and Ukraine. Moreover, in various countries, the respondents suggested that improvement of communication strategies and specific information programs might be crucial to reach vaccine-hesitant citizens and facilitate the vaccine rollout.

In addition to traditional media, social media were noted to play a role. Social media influencers were identified to positively contribute to motivating people to be vaccinated, producing a “crowd effect,” as reported for Croatia and Ukraine, where public figures, such as the President and the health minister gave declarations through the media. Vivid promotions in favor of vaccination by persuasive political and medical discourses, accompanied by enthusiastic argumentations in favor of science and against conspiracy theories and vaccination skepticism, were described for Slovenia.

#### Threats

The primary themes related to threats were as follows: (1) appearance of new virus strains/lower efficacy of the vaccines, (2) unforeseen side effects of the vaccines, (3) spreading disinformation, (4) ethical controversies, (5) legal controversies, (6) religious controversies, and (7) a change in the behavior of vaccinated individuals that facilitates the spread of infection.

Low trust in the efficacy and safety of the vaccines (Romania, Slovenia, Ukraine); a negative influencing role played by some media communications, especially when stressing the side effects (Serbia, Sweden), and alleged corruption related to the vaccine prioritization (Slovenia) were regarded as relevant threats to be considered for achieving successful mass vaccination campaigns. Insufficient information, disinformation, or misinformation in the media and on the internet were reported for the Czech Republic, Sweden, and Romania, while development of conspiracy theories about vaccines was pointed out for Slovenia and Ukraine. Disputable communication from the government regarding vaccines and other public health measures such as lockdowns was described for Germany. Lack of adequate public communication strategies was also noted in Slovenia. Failures in communication with people from different cultural groups were reported in Australia.

Ethical concerns associated with the use of leftover doses were pointed out by respondents from Sweden and Portugal, referring to a lack of planning for how to handle leftover vaccines that could not be administered the next day or to the overall mismanagement of vaccine administration. In contrast, the opportunity to get a leftover dose was marked as a strength at the beginning of the vaccination campaign in Ukraine, where this was the only option to be vaccinated for those in nonpriority groups. Confusing messages from religious leaders and local community priests were reported in Romania. Concerns of disobeying the Islamic conduct codes raised by vaccination opponents was described for Turkey, as during the month of Ramadan fears were prompted that vaccination during the fasting period was not acceptable.

### Frequency Analysis

#### Overview

**Figure 2:**
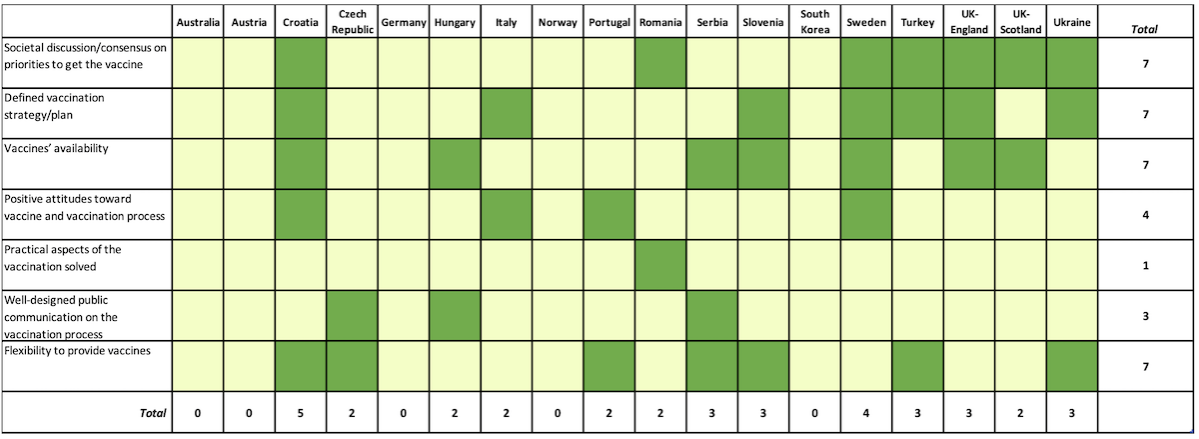
Overview of the opinions covering strengths-related themes by country. Green indicates presence of a theme (assigned a value of 1) and yellow indicates absence of the theme (assigned a value of 0). The total score corresponds to the sum of values of all related responses.

**Figure 3:**
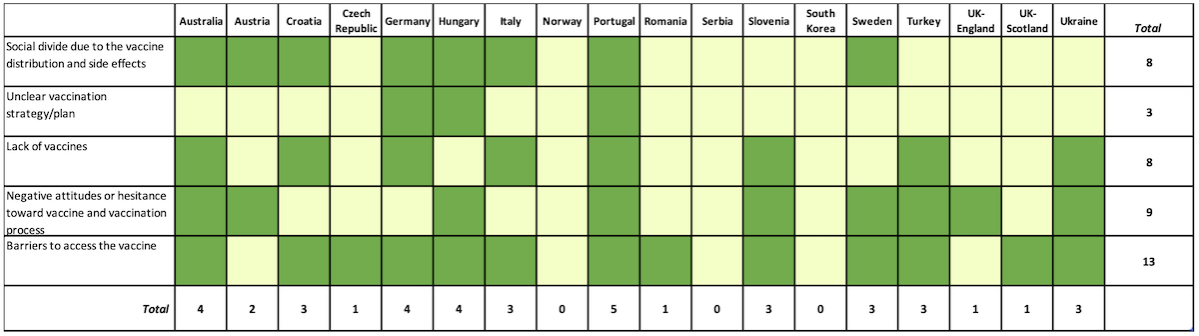
Overview of the opinions covering weaknesses-related themes by country. Green indicates presence of a theme (assigned a value of 1) and yellow indicates absence of the theme (assigned a value of 0). The total score corresponds to the sum of values of all related responses.

**Figure 4:**
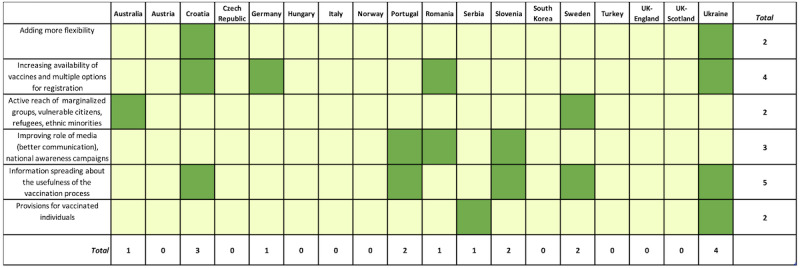
Overview of the opinions covering opportunities-related themes by country. Green indicates presence of a theme (assigned a value of 1) and yellow indicates absence of the theme (assigned a value of 0). The total score corresponds to the sum of values of all related responses.

**Figure 5:**
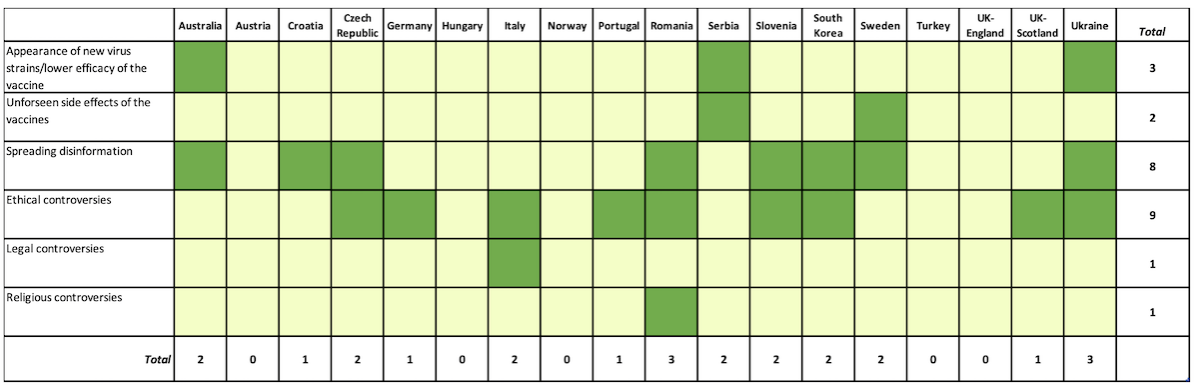
Overview of the opinions covering threats-related themes by country. Green indicates presence of a theme (assigned a value of 1) and yellow indicates absence of the theme (assigned a value of 0). The total score corresponds to the sum of values of all related responses.

To explore the distribution of the responses by countries, frequency analysis was performed (Figures 2-5). Responses reporting a certain theme are marked in the figures in green color and assigned a value of 1, whereas those that did not mention the theme are marked with light yellow and assigned a value of 0. The total score corresponds to the sum of values of all related responses. Additionally, the average percentage of responses distributed for each element and theme was calculated (Multimedia Appendices 2-5).

#### Strengths

Three themes dominated the analysis of strengths, each being covered in 7 reports: societal discussion/consensus on priorities to get the vaccine, defined vaccination strategy/plan, vaccines’ availability, and flexibility to provide vaccines.

The strengths theme “societal discussion/consensus on priorities to get the vaccine” was mentioned in Croatia, Romania, Sweden, Turkey, UK Scotland, UK England, and Ukraine. “Defined vaccination strategy/plan” was reported in Croatia, Italy, Slovenia, Sweden, Turkey, UK (England), and Ukraine. “Wide vaccines availability” was indicated in Croatia, Hungary, Serbia, Slovenia, Sweden, and the United Kingdom. “Positive attitudes of the society toward vaccines and vaccination process” were reported for Croatia, Italy, Portugal, and Sweden. Logistic aspects of the vaccination being solved (including satisfaction of the medical personnel, sites easy to access for registration, fast process, no waiting in line) were noted for Romania, while well-designed public communication on the vaccination process was described for the Czech Republic, Hungary, and Serbia. Flexibility to provide vaccines was highlighted as a potential strength in the contributions from Croatia, Czech Republic, Portugal, Serbia, Slovenia, Turkey, and Ukraine. Relatively even distribution was identified across strengths categories with the exception of practical aspects of the vaccination solved that was reported by only one contributor.

#### Weaknesses

Most frequently reported weaknesses were barriers to access the vaccination (13 reports) and negative attitudes or hesitancy toward vaccines and the vaccination process (9 reports).

Social divide due to the vaccine distribution and side effects were considered weaknesses in Australia, Austria, Croatia, Germany, Hungary, Italy, Portugal, and Sweden. Unclear vaccination strategy/plan was described in Germany, Hungary, and Portugal. Lack of vaccines was noted in Australia, Croatia, Germany, Italy, Portugal, Slovenia, Turkey, and Ukraine (note that the questionnaire addressed these issues only related to the first 6 months of the vaccination campaigns). Negative attitudes or hesitancy toward vaccines and the vaccination process were described in Australia, Austria, Hungary, Portugal, Slovenia, Sweden, Turkey, UK England, and Ukraine, while barriers to access vaccination, including problems with prioritization, registration, and unfair/nontransparent distribution of the vaccines were noted in Australia, Croatia, Germany, Hungary, Italy, Czech Republic, Portugal, Romania, Slovenia, Sweden, Turkey, UK Scotland, and Ukraine. In addition, 72.2% of the contributions reported barriers to access vaccines as a weakness. Conversely, only 16.6% of our sample reported an unclear vaccination strategy/plan as weakness.

#### Opportunities

The frequencies of the selected opportunities to improve the vaccination process were rather low including a maximum of 5 countries. Adding more flexibility to the vaccination process was mentioned in Croatia and Ukraine; increasing availability of the vaccines and multiple options for registration were mentioned in Croatia, Germany, Romania, and Ukraine; active reach of marginalized groups, vulnerable citizens, refugees, and ethnic minorities was mentioned in Australia and Sweden; improving the role of the media (better communication) and national awareness campaigns were indicated in Portugal, Romania, and Slovenia; information spreading about the usefulness of the vaccination process was highlighted in Croatia, Portugal, Slovenia, Sweden, and Ukraine; and monetary provisions for vaccinated individuals were mentioned in Serbia and Ukraine. Contributors did not report opportunities in large numbers. The highest percentage (27.7%) of responses related to the opportunities-related themes was attributed to spreading information about the usefulness of the vaccination process.

#### Threats

Concerning the possible threats to a vaccination campaign’s success, the contributors from Australia, Serbia, and Ukraine remarked the possible appearance of new virus strains and lower efficacy of the vaccine; unforeseen side effects were noted as possible threats in the contributions from Serbia and Sweden; spreading disinformation were noted or could be concluded from the abstracts from Australia, Croatia, Czech Republic, Romania, Slovenia, South Korea, Sweden, and Ukraine. Other possible threats to vaccination success mentioned were ethical (Czech Republic, Germany, Italy, Portugal, Romania, Slovenia, South Korea, UK Scotland, and Ukraine), along with legal (Italy) and religious controversies (Romania). Ethical controversies (50%) and spreading information (44.4%) were the most highly represented threats-related themes.

In this study, the mainly acknowledged threat feature for achieving successful vaccination campaigns reported by the respondents was related to the likely occurrence of viral mutations, resulting in new virus strains with the ability to escape the immunizing effects of the present available vaccines. This fact has been pointed out as a relevant source of uncertainties and doubts about the vaccines’ effectiveness as well as about their overall reliability and utility.

## Discussion

### Principal Findings

Since its introduction in the 18th century to the present day, vaccination has been one of the most effective tools in the battle against infectious diseases [10,43]. Owing to the high efficacy of vaccines, the public health burden of infectious diseases has been significantly reduced throughout the years [10]. However, despite their proven track record, the phenomenon known as “vaccine hesitancy” has been around almost as long as vaccination itself. This reluctance to accept an injection of an “unknown substance” into the body is exasperated by a need to vaccinate a large number of healthy individuals, including in the case of COVID-19 [44].

**Figure 6:**
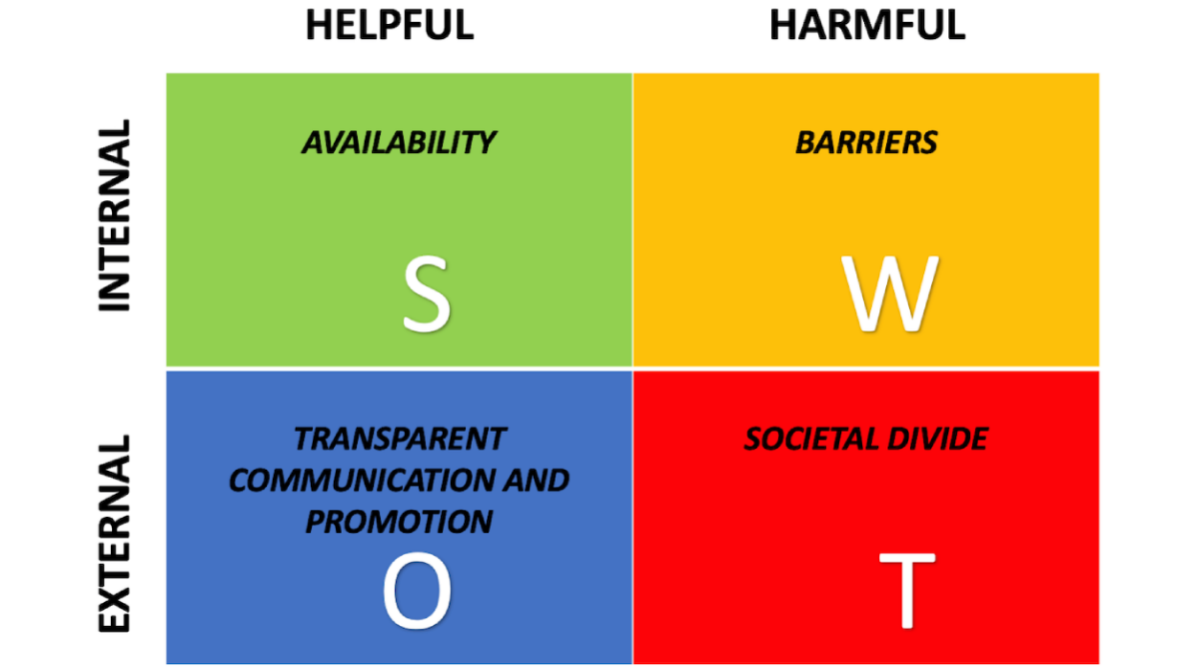
Synthesized themes under the strengths (S), weaknesses (W), opportunities (O), and threats (T) framework.

This study, based on an analysis of interdisciplinary experts’ viewpoints in 17 different countries inspired by the SWOT framework, allowed us to identify 25 themes distributed across the four SWOT elements. To our knowledge, this is the first study to analyze and compare the vaccine rollout process in various different countries. The frequency of the appearance of these themes and their distribution across the countries allowed us to select those that stand out. As the contributions were inspired by the SWOT framework, the presented analysis could be easily synthesized into the four main overarching SWOT categories (Figure 6). With respect to the strengths of the vaccination process, the identified seven themes correspond to a single category referred as *Availability*, being the major strength of the successful vaccination program. When weaknesses were described, the five themes identified could be best described by a single category termed *Barriers*, which were either not recognized or not addressed by the vaccination programs*.* The external aspects of opportunities described via the six themes identified fit under category *Transparent communication and promotion,* which allows other societal forces to contribute to the vaccination process. Finally, the seven themes describing threats correspond to the *Societal divide* category, where a polarized society has the potential to spoil even well-thought-out initiatives.

We believe that these categories offer the best representation of the most frequently reported themes in each of the SWOT elements. However, due to the intertwining factors present in the vaccination rollout process, it is important to not look at this distribution as a binary (presence/absence) phenomenon. This is particularly relevant when splitting the identified themes into “internal” and “external” categories. In the current SWOT 2×2 matrix (Figure 6), *Availability* and *Barriers* are labeled as internal categories, whereas *Transparent communication and promotion* and *Societal divide* are suggested as external categories [45]. However, within the *Societal divide* category labeled as a threat, there are ethical, religious, and legal controversies reported as important themes. Therefore, one cannot classify a controversy per se as a threat, as controversies can serve just as much as a source of debate with the potential to improve the vaccination process.

The specific time window when the study was performed corresponds to a relatively early phase of the vaccination process (on average half of the population had been vaccinated in the analyzed countries). This leads to a very specific bias in the submitted data: urgency to tackle an important and pressing issue. We reiterate that our study analyzed the subjective viewpoints of the respondents; hence, some of the themes across these categories were dependent on the various individual, psychological, emotional, and societal aspects specific to the given time window. The sudden appearance of COVID-19 and its rapid spread called for appropriately rapid responses. Considering psychological factors of egocentrism, information availability, social/group confirmation, individual motivation, and emotional affect as foundations of that rapid decision-making process, it is easily possible to misjudge and/or misperceive the key elements of the reasoning arising from the complexity of the situation [46]. However, although there were 27 individual respondents from 17 different countries, our results did not show country-specific differences. Hence, our findings can contribute to the development of strategies that will maximize the promotion of strengths and opportunities while minimizing weaknesses and threats globally.

The identified themes are consistent with the research on this topic [36,38]. The most prominent theme in the existing literature, which was also present in our study, explores the effective medical and public health system measures mapping on the key strengths identified herein. This shows how preparation and prevention strategies work, and how they can be used as a base of the powerful pushback against the spread of COVID-19. Moreover, a positive attitude toward vaccination has been defined as a strength in similar studies in India and Zimbabwe [36,38].

The application of the SWOT framework to complex societal processes can also be seen as a source of confusion. For example, a “strength” is considered as an internal aspect of the process, which can be understood to relate to the vaccination campaign itself. From this perspective, the attitude toward vaccination does not seem to be an internal component but rather an external aspect of SWOT and thus should be more appropriately classified as an opportunity rather than a strength. However, application of the SWOT framework in such complex scenarios requires consideration of the vaccination campaign as part of a sociotechnical system, thus incorporating vital elements of the social environment within the situated practice of vaccination. Combining our findings obtained from individuals from 17 different countries with previous research, it can be concluded that good organization that addresses the availability of vaccines coupled with an engaging societal discussion would represent a key strength/opportunity of the vaccination process.

A lack/shortage of vaccines combined with various logistical challenges have been reported as major issues for the success of vaccination campaigns within previous research [47,48]. The demand-supply gap combined with lack of knowledge and supporting infrastructures have been reported as particular weaknesses [36,38]. Compounding unequal vaccine distribution with unknown disease progression and an uncertain response to the vaccine seems to be the biggest barrier in the vaccine rollout [13]. Similarly, the respondents of this study recognized the practical issues of availability and fairness of distribution, and coupled these issues with the related attitudes and social division. This points to the fact that social distrust needs to be addressed within a vaccination plan as a major barrier. For both strengths and weaknesses, no clear geographical divide was present.

Increasing the public awareness about the vaccine effects through transparent communication and promotion stood out as a key opportunity-related theme. Communication reports on the widespread acceptance of COVID-19 vaccines have shown to be effective tools to further increase vaccine acceptance [49]. Moreover, in an attempt to promote vaccination, some public figures have been vaccinated on television [49]. It is interesting to see that people who used mainstream media outlets as their major source of information on health were more likely to get vaccinated [50]. Our data support the notion that transparent information-sharing about biological mechanisms, efficacy, as well as side effects of the vaccines motivates people to join vaccination programs. Previous research has identified the potentially influential role of media in increasing people’s trust in vaccines when they hear politicians, celebrities, or other famous people talking positively about them [36]. Trust in vaccines, medical science, and medical professionals—together with other involved stakeholders, including government and policy makers—was highlighted in the analyzed contributions as an important factor. These findings align with previous research that found lack of communication from trusted providers and community leaders as one of the main reasons for low COVID-19 vaccination rates [44]. Communication of vaccine information and promotion of its uptake in the digital era includes the use of social networks [51]. However, the use of social networks is also associated with risks due to the wildfire-like dynamics of rumors in the digital environment and issues with unknown algorithms used by for-profit entities filtering information [52,53]. Social networks are expected to drive healthy public debates; however, they instead frequently reinforce like-minded “bubbles” and increase polarization [53,54].

Discussions on matters of autonomy (an individual’s right to choose) and state power have always been at the center of public health ethical dilemmas [29]. In the specific case of COVID-19 vaccines, besides the tensions between public health and individual interest/autonomy, other ethical challenges relate to the rapid design and testing of vaccines and who gets the vaccines (first) [55]. Public health authorities need to implement efficient, flexible, responsive, and resilient strategies to successfully fight the pandemic and raise awareness of all of the dangers arising with this disease [56]. Surprisingly, in our findings, the question of one’s autonomy did not crystalize as a theme. Instead, other ethical controversies and spreading of disinformation were found to be the most frequently reported themes within the threats element. In the present digital era, information accessibility is at its peak; however, it is important be aware of the source of the information given that rumors and fake news are rampant [17,57].

When discussing the threats element in the SWOT framework, it was interesting that unforeseen side effects of the vaccine have not been considered as the most prominent threat theme, whereas other research shows that the most common reason for vaccine hesitancy or refusal is due to the concerns related to the side effects/safety [50,58,59]. The emergence of new virus strains was mentioned as a threat, since they decrease the efficacy of the vaccine and hence can contribute to the further spread of COVID-19. As people were already worried about the lack of information about safety, testing, and efficacy of COVID-19 vaccines, the new variants were seen as a contributor to the negative perception of the vaccines in society [15,60]. The synthesizing category for the threat element of *Societal divide* implies that social polarizations have the potential to paralyze a society when facing a complex public health crisis. Here, it should be stated that silencing the controversies is certainly not the path to avoid such an outcome. A society where controversies are not openly discussed is not without these controversies, but rather this situation would give rise to potentially dangerous and isolation subcultures. Although *Societal divide* was recognized as a threat in our sample, there were no clear examples where this has significantly directly influenced the vaccination process. Consequently, although the awareness of controversies as a potential threat was voiced, if the social environment is developed within the context of *Transparent communication and promotion* (opportunity), the *Social divide* may never reach the level of polarization to create adverse effects on public health campaigns.

### Study Limitations

The collected responses represent the subjective viewpoints of experts who volunteered to take part in the study. Therefore, extrapolation to the national level must be drawn out with caution. In addition, due to the lack of research using this same methodology and implementing it on a multinational level, there were no relevant studies to make direct comparisons with and contrast conclusions. Moreover, SWOT analysis was not performed in its original form addressing organizational dynamics. Instead, this thematic analysis of expert viewpoints was only inspired by the SWOT framework. Therefore, the results of this study should be further examined and more research is needed on this topic in general. Further studies could consider interdisciplinary and multinational frameworks to find the best practice in public health policies that could yield improved vaccination rollout results globally.

### Conclusion

This study was based on a collection of short responses to a specifically designed questionnaire, written by researchers from different countries and fields of expertise, thus bringing together multidisciplinary and cross-national opinions on vaccination rollout. This represents the first analysis of the vaccination process in 17 different countries inspired by the SWOT framework. The obtained results highlight the connection between organizational aspects of the vaccination rollout and corresponding societal response, both being related to the strengths and weaknesses of the process. The opportunities and threats corresponded to external societal factors, better public communication of vaccination-related issues, ethical controversies, and the spread of disinformation. The inventory of 25 SWOT-related themes and the resulting 2×2 SWOT matrix represents an approximate best-practice viewpoint for the successful implementation of public health policies—as represented by this multidisciplinary team—in the fight against COVID-19.

## Appendix

Multimedia Appendix 1 Share of people from countries in our sample who received at least one dose of COVID-19 vaccine (taken from Our World in Data. Coronavirus (COVID-19) Vaccinations. 2021 [42]).

Multimedia Appendix 2 Average percentage of opinions covering strengths-related themes.

Multimedia Appendix 3 Average percentage of opinions covering weaknesses-related themes.

Multimedia Appendix 4 Average percentage of opinions covering opportunities-related themes.

Multimedia Appendix 5 Average percentage of opinions covering threats-related themes.

## Abbreviations

NKL: Navigating Knowledge Landscape
SWOT: strengths, weaknesses, opportunities, and threats

## Data Availability

The data sets supporting the results presented in this study can be found in the online repository [39].
