# Supplementary figures and images for: Perspectives on the COVID-19 Vaccination Rollout in 17 Countries: Reflexive Thematic and Frequency Analysis Based on the Strengths, Weaknesses, Opportunities, and Threats (SWOT) Framework
Source: JMIR Hum Factors. 2024 Feb 19;11:e44258. doi: 10.2196/44258 (PMC10896317; doi:10.2196/44258)

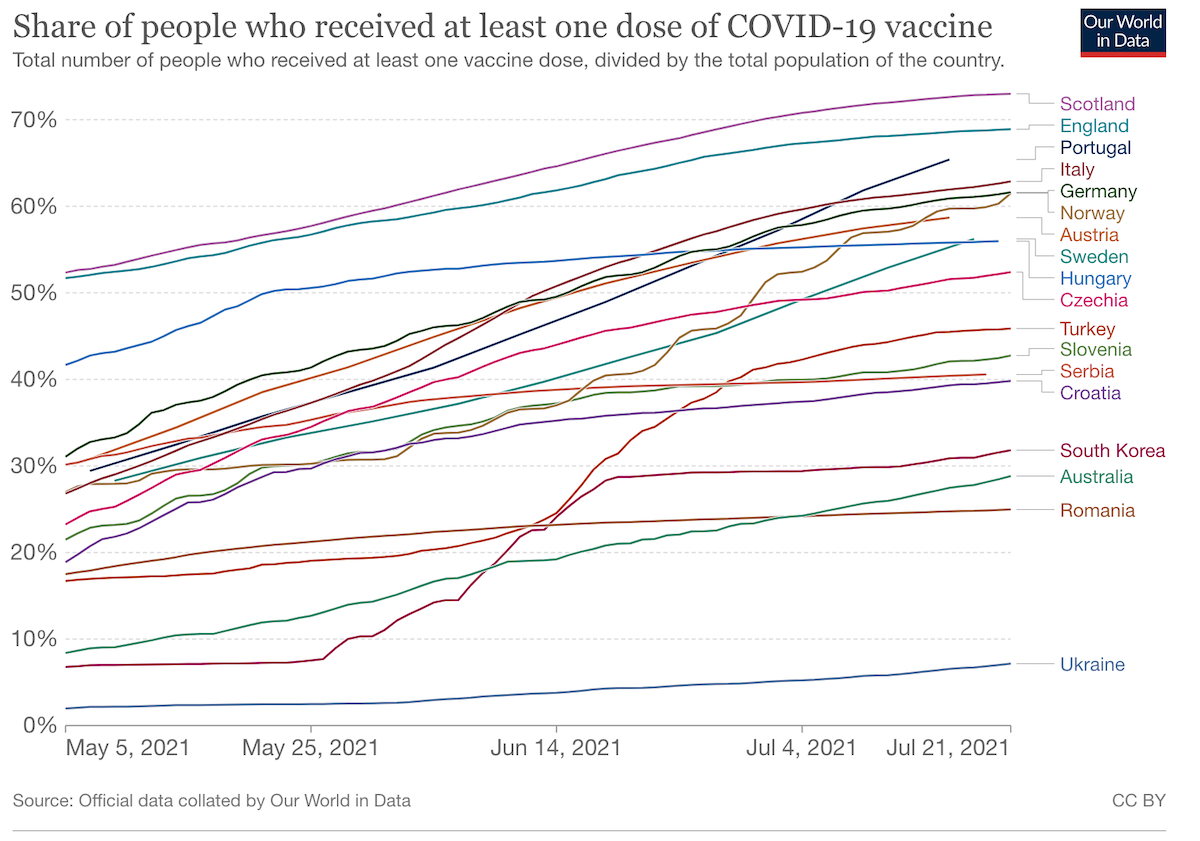

Supplement: Multimedia Appendix 1 [file humanfactors_v11i1e44258_app1.png]

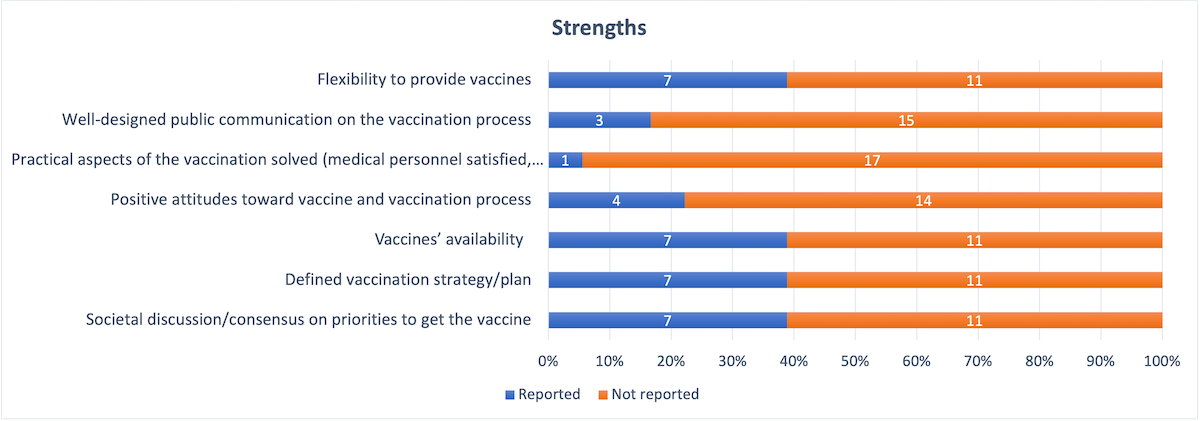

Supplement: Multimedia Appendix 2 [file humanfactors_v11i1e44258_app2.png]

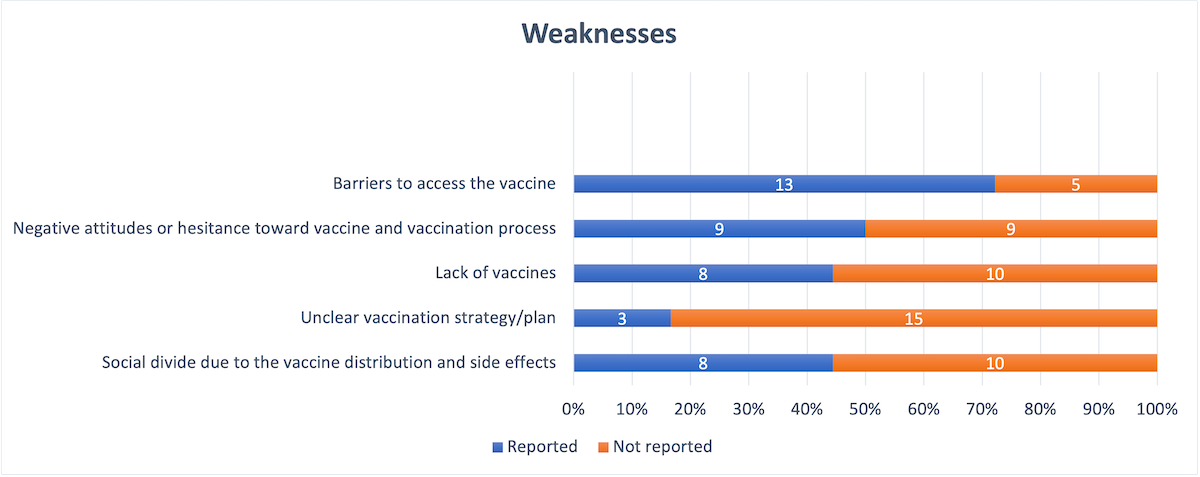

Supplement: Multimedia Appendix 3 [file humanfactors_v11i1e44258_app3.png]

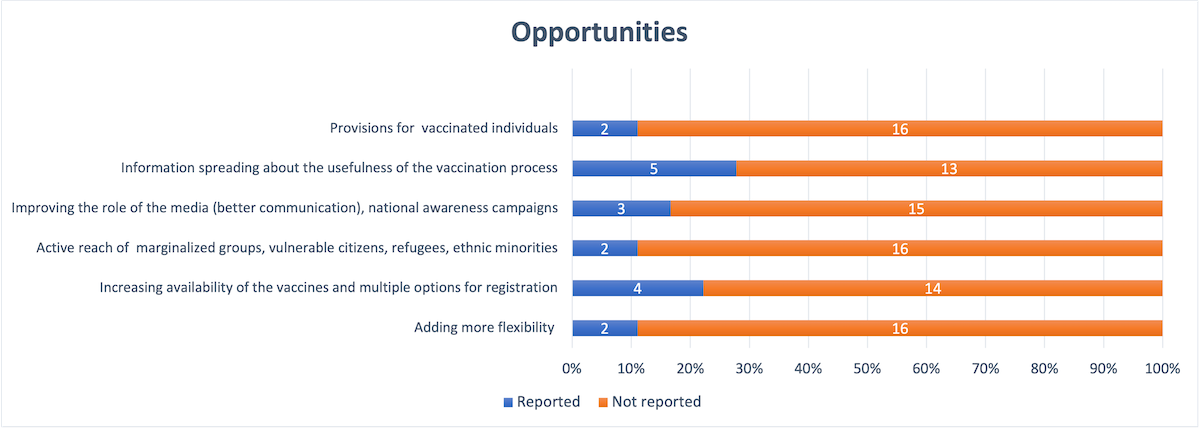

Supplement: Multimedia Appendix 4 [file humanfactors_v11i1e44258_app4.png]

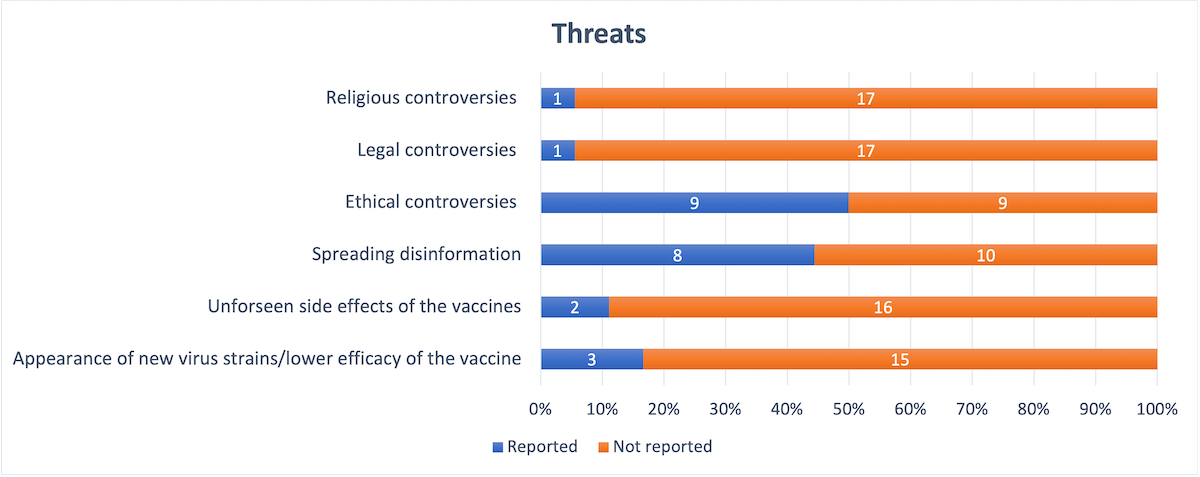

Supplement: Multimedia Appendix 5 [file humanfactors_v11i1e44258_app5.png]
